# A Bioimpedance-Based Device to Assess the Volume Conduction Properties of the Tongue in Neurological Disorders Affecting Bulbar function

**DOI:** 10.1109/OJEMB.2021.3117871

**Published:** 2021-10-06

**Authors:** Xuesong Luo, Hilda V. Gutierrez Pulido, Seward B. Rutkove, Benjamin Sanchez

**Affiliations:** Department of Neurology, Beth Israel Deaconess Medical CenterHarvard Medical School 1811 Boston MA 02115 USA; Sanchez Research Lab, Department of Electrical and Computer EngineeringUniversity of Utah 7060 Salt Lake City UT 84112 USA

**Keywords:** Tongue, volume conduction properties, anisotropy, electrodes, biomarker

## Abstract

*Goal:* Current instruments for bulbar assessment exhibit technical limitations that hinder the execution of clinical studies. The volume conduction properties (VCP) of the tongue reflect ionic content and myofiber integrity and they can serve as a new biomarker for evaluating neurological disorders with bulbar dysfunction. *Methods:* We designed a standalone bioimpedance measurement system that enables accurate, multi-frequency measurement of tongue anisotropic VCP including conductivity and relative permittivity. The system includes a tongue depressor with 16 non-invasive surface sensors for electrical contact with the tongue at directions 0$^{\circ }$, 45$^{\circ }$, 90$^{\circ }$ and 150$^{\circ }$. The depressor is interfaced with the tongue electronic system with Bluetooth connectivity, and a smartphone application. De-identified patient data is sent by email. *Results:* We first determined the accuracy of the hardware performing phantom measurements mimicking a broad range of tongue values and determined the error to be $< $1%. We then validated our new technology measuring a cohort of 7 healthy human subjects under Institutional Review Board approval. *Conclusions:* None of the subjects who participated suffered discomfort or gag reflexes. The novel technique presented for intra-oral assessment of tongue VCP provides standard, objective and quantitative data potentially sensitive to alterations in tongue internal structure and composition.

## I. Introduction

Speech and swallowing abnormalities accompany many neurological disorders. In amyotrophic lateral sclerosis (ALS) for example, approximately 25% of patients have predominantly such complaints, termed bulbar symptoms, at the time of disease onset [1]. In addition to ALS, there are many other neuromuscular disorders (NMD) that deteriorate tongue health in both children and adult people including myasthenia gravis and oculopharyngeal, facioscapulohumeral (FSHD), and Duchenne muscular dystrophies [2], [3]. Outside the realm of the NMD, Parkinson's disease, Huntington's disease, and cortical and cerebral white matter injury, due for example, to cerebrovascular and demyelinating disease also reduce tongue function thus impacting the ability to communicate, eat, and swallow effectively [4].

Patients with speech and swallowing dysfunction require a holistic integration including neurologists, otolaryngologists, speech-language pathologists, each with their own evaluation procedures and assessment tools. While history and examination must guide the use of investigative studies and treatment, subclinical bulbar involvement is common. In ALS in particular, the Revised ALS-Functional Rating Scale (ALSFRS-R) bulbar sub-score is the only measure routinely used to evaluate bulbar dysfunction in the clinical setting [5]. The ALSFRS-R test includes a total of 3 questions (out of 12 in the total ALSFRS-R) to rate the patient's level of bulbar impairment in performing speech, salivation, and swallowing tasks. Each task is then rated on a five-point coarse scale from 0 = can’t do, to 4 = normal ability. Individual item scores are summed to produce a bulbar sub-score of between 0 = worst and 12 = best. The facial disability index (FDI) is another questionnaire available to assess facial function, where the total score is transformed onto a percentage scale, with 100 representing normal and lower scores representing increasing disability. While clinically valuable, ALSFRS-R and FDI have limited assessment of bulbar dysfunction which may lead to delayed bulbar impairment assessment and underestimation of disease severity. In the end, other modalities are needed to support facial and bulbar sub-scores, discussed below.

Video fluoroscopic swallowing exam (VFSE) allows the practitioner to visualize in real time patient's ability to swallow safely and effectively various types of barium-containing foods, but the level of inter-rater agreement is poor [6], [7]. Further, VFSE does not provide quantifiable metrics, requires the ability of the patient to cooperate effectively which can be difficult to achieve in children and subjects with cognitive impairment, examiner training, expensive equipment, and x-ray exposure [6], [7]. Needle electromyography provides useful evidence of neuron loss in patients with ALS but requires considerable expertise, is painful, and cannot be readily quantified [8], [9]. Quantitative tongue pressure manometers have been proposed [10], but these also require the patient to be able to cooperate. Also, tongue pressure values are dependent on subject's motivation, number of trials, patient feedback, and tongue and jaw position [11]. Importantly, these techniques are all inconvenient, require considerable practice in performing consistently, making them challenging to employ on a regular basis to detect subtle deterioration.

**FIGURE 1.**
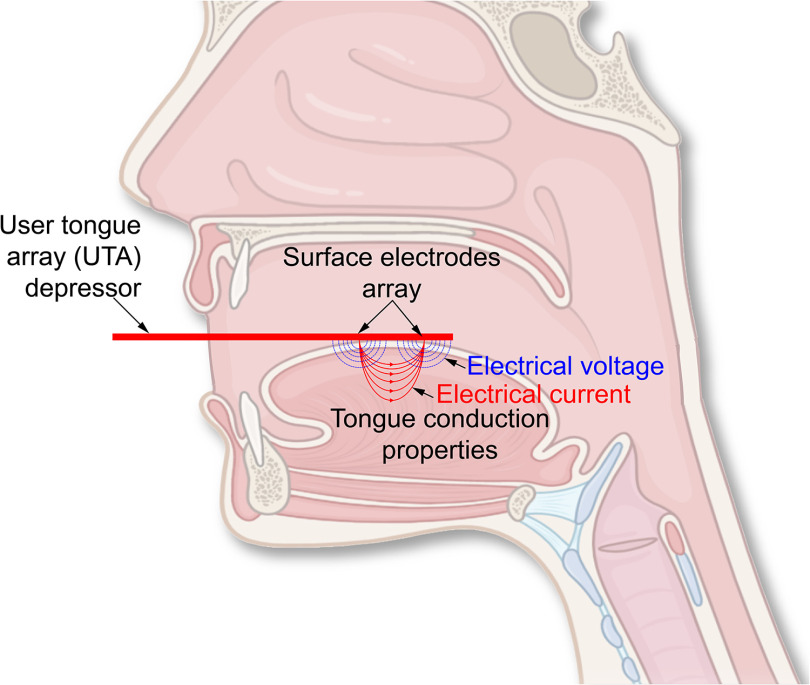
Illustration of the tongue depressor technology developed for in vivo assessment of tongue volume conduction properties (VCP). The test measures tongue VCP data using two electrodes the voltage resulting from applying a non-stimulating high-frequency electrical current across a region of tongue using a different pair of electrodes. We refer the reader to [13] for further simulation details of the actual measured with the tongue depressor.

Despite the value of these existing tools, innovative technologies could offer revolutionary solutions to the current gap in measures of bulbar dysfunction. For example, in patients with FSHD, orofacial muscles including the tongue are progressively affected. Specifically, the tongue muscle fibers atrophy, gradually being replaced by interstitial fat and connective tissue. These pathological changes impact the intra- and extra-cellular ionic concentrations and myofiber membrane integrity of the tongue, altering its ability to conduct electrical current and store electric charges, characteristics termed volume conduction properties (VCPs) [12] (see Fig. 1).

**FIGURE 2.**
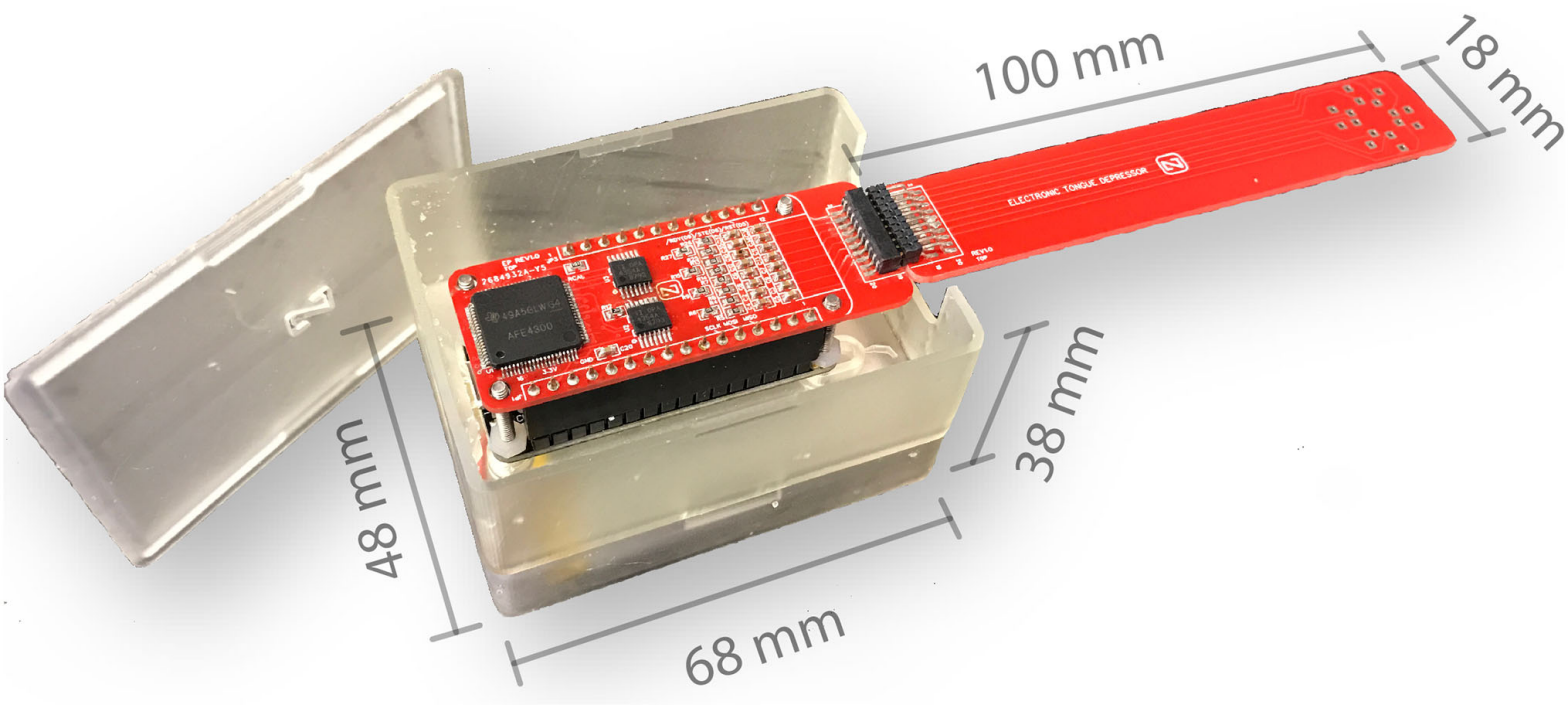
Picture of the user tongue array (UTA) developed plugged into the user tongue electronic system (UTES).

Here, we present a novel hardware development for intra-oral tongue VCP assessment based on a low-cost, open source hardware Internet of Things (IoT) platform (see Fig. 2). The ease-of-use of the technology implemented around IoT device may provide an optimal solution for at-home application, thus allowing ALS or FSHD patients to participate in drug trials taking measurements on themselves far more frequently than would be possible visiting a medical center [14], [15]. Below we provide a description of our technology, preliminary phantom testing on circuits, and finally its successful application measuring healthy subjects.

## II. Materials and Methods

**FIGURE 3.**
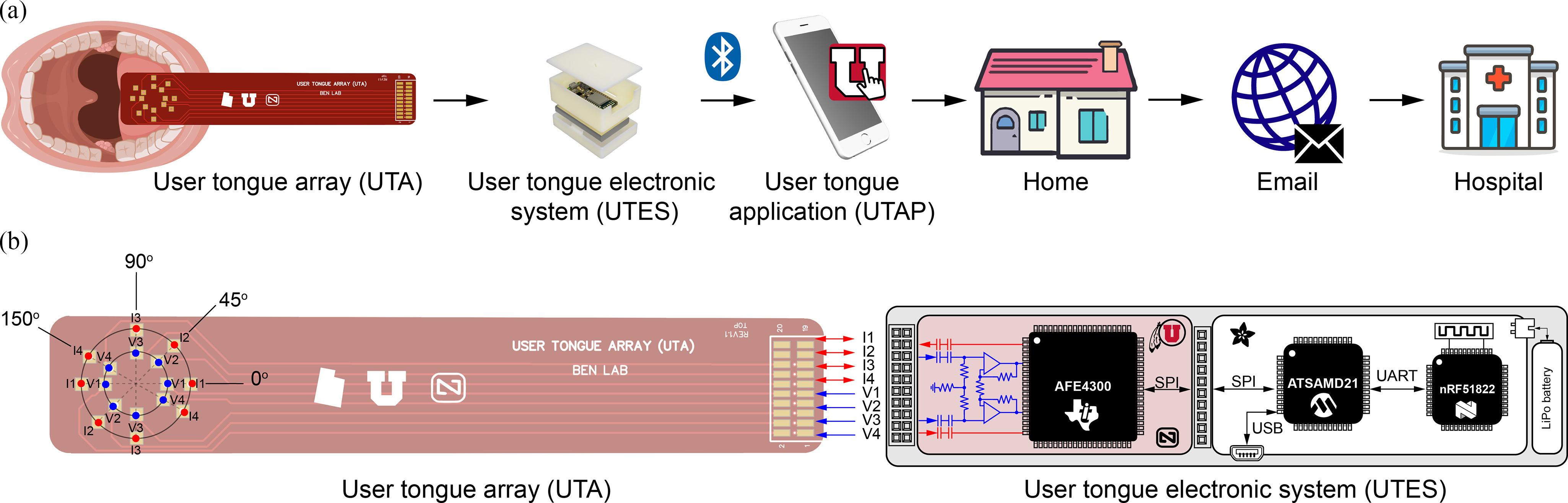
(a) Diagram showing a tongue measurement using the user tongue array (UTA) depressor. The UTA connects to the user tongue electronic system (UTES), which performs directional tongue measurements. Data is sent from UTES to a custom-built Android user tongue application (UTAP) installed on a smartphone via Bluetooth. Finally, de-identified tongue data are sent via email to the hospital. (b) Schematic of the hardware architecture developed. The UTES includes the development board Feather M0 Bluefruit LE (Adafruit, Inc., New York City, NY), a custom analog front end (AFE) board for impedance measurement, and a lithium polymer (LiPo) battery. The UTA depressor connects to the AFE board for measuring the tongue in four directions determined by the angles 0$^\circ$, 45$^\circ$, 90$^\circ$ and 150$^\circ$ (in red and blue color). Abbreviations: serial peripheral interface, SPI; universal serial bus, USB; universal asynchronous receiver/transmitter, UART.

Fig. 3(a) shows an overview of the architecture of the developed system as well as its intended functionality.

### A. User Tongue Array (UTA) Depressor

The user tongue array (UTA) depressor is a printed circuit board (PCB) of standard dimensions length 100 mm × width 18 mm. On the tongue contact side, 16 contacts are arranged in two concentric circles of radii 4 and 7 mm, respectively; and measuring directions determined by the angles 0$^\circ$, 45$^\circ$, 90$^\circ$ and 150$^\circ$. The electrodes connect directly to the expansion connector located in at the connection side (see Fig. 3 b). The UTA is fully manufactured with standard PCB manufacturing process, it is disposable and can be sterilized using isopropyl alcohol. The electrodes are soldering points using solid Pb-free soldering paste directly soldered on the side of the depressor in touch with the tongue.

### B. User Tongue Electronic System (UTES)

1) **Hardware:** The user tongue electronic system (UTES) hardware (see Fig. 3 b) is based on Adafruit IoT development platform Feather M0 Bluefruit LE. The dimensions are length 51 mm × width 23 mm × height 8 mm, including the ATSAMD21G18 ARM Cortex-M0+ processor (Microchip, Inc., Chandler, AZ) and Bluetooth low energy nRF51822 module (Nordic Semiconductor, Inc., Trondheim, Norway). The microprocessor is programmed directly via USB with the Arduino IDE software environment. The Feather M0 board has built in the power management and battery charging circuitry and is powered with an external 3.7 V and 2200 mAh lithium polymer battery.

Tongue VCP measurements are performed with a custom analog front end (AFE) board connected on the Feather's expansion connectors (see Fig. 2). The AFE is based on the integrated circuit AFE4300 (Texas Instruments, Inc., Dallas, TX) [16], which contains the current generating source, multiplexer, demodulating circuit, and analog to digital converter circuitry. The AFE4300 measures the tongue VCP including the conductivity and relative permittivity values by applying a stepped-sine excitation at (8, 16, 32, 64, 128 and 256) kHz with amplitude 0.1 mA$_{\rm rms}$ [17] The voltage generated between the current electrodes as a result of the anisotropic conduction properties of the tongue is then measured in all four directions. For each direction, the external circuitry consists of DC-blocking capacitors, T-resistive networks, operation amplifier based buffers (OPA4354, Texas Instruments, Inc.), and then measured with the internal differential amplifier in the AFE4300. Directional tongue data are sent to the microprocessor via serial peripheral interface (SPI) protocol for data processing.

The UTES housing was designed in Fusion 360 (Autodesk, Inc., San Rafael, CA) with a minimum form factor (length 68 mm × width 48 mm × height 38 mm) while allowing to program the microprocessor through USB and to easily connect the UTA. The housing was created using 3D printing technology with photopolymer resin using (Form 3, Formlabs, Inc., Somerville, MA). The housing consists of three separate parts: battery holder, case, and topper case. Internal cable routing connects the battery to the Feather board and the user can turn the UTES on or off with a side switch in the battery holder.

2) **Firmware:** The firmware starts up upon power up of UTES by initializing the AFE board and the Bluetooth radio chip via SPI. Then micro-controller is waiting to build communication. Once finished, the microcontroller awaits in idle state for a valid communication link through Bluetooth using the UTAP or universal asynchronous receiver/transmitter (UART) protocol via the USB connector. The UART link is for system maintenance, debugging and calibration using a computer. The calibration parameters for all channels and frequencies are calculated and stored in the firmware through an automated sequence [18]. Switching working modes is achieved through a compilation directive. Next, a command interpreter performs data collection upon request. Once the connection is established and the user has placed the sensors of the UTA depressor touching the tongue, the user can perform a multi-directional tongue impedance measurement. A reconstruction method (see Supplementary Materials) is then implemented for calculating anisotropic tongue VCP including the conductivity and the relative permittivity [19], [20].

### C. User Tongue Application (UTAP)

**FIGURE 4.**
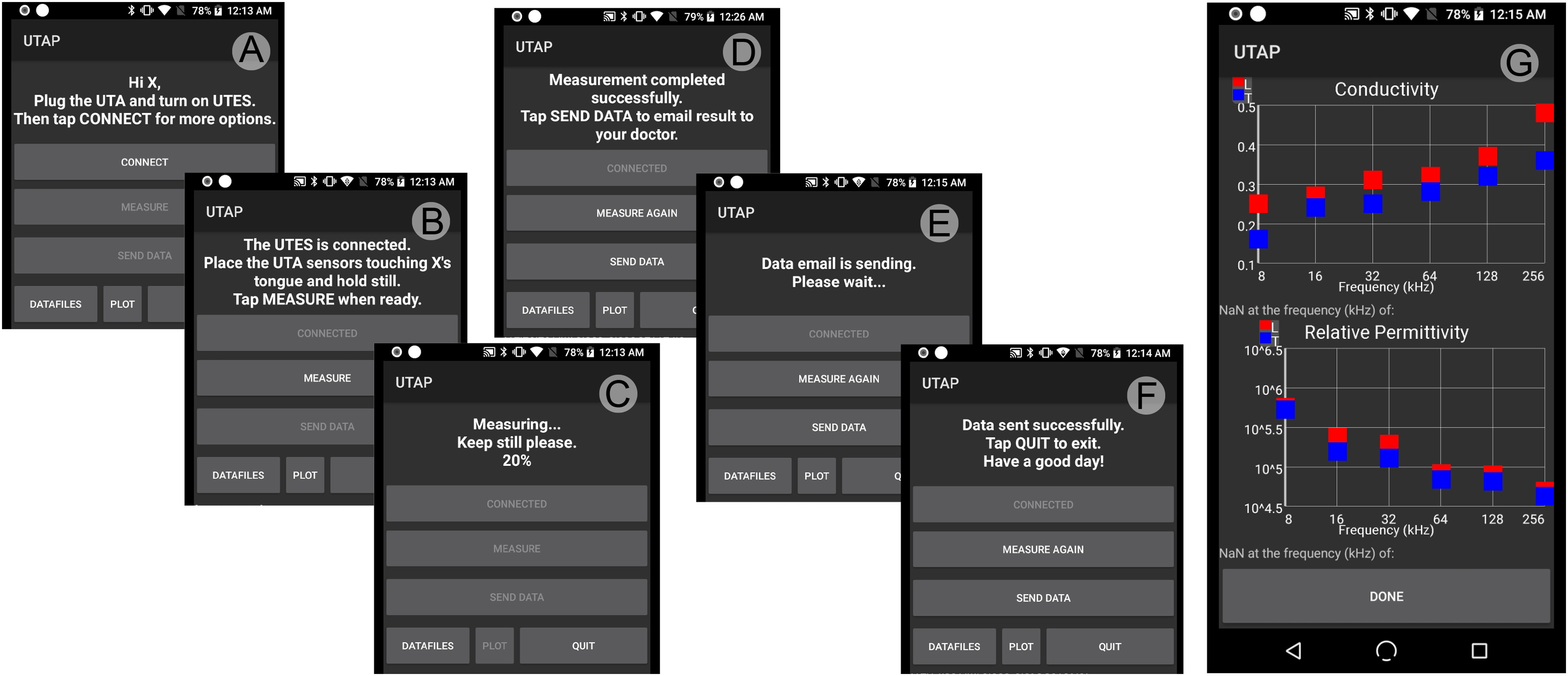
User tongue application (UTAP). The UTAP connects to the user tongue electronic system (UTES) via Bluetooth (A). Once the connection is established and the user has placed the sensors of the user tongue array (UTA) touching the tongue (B), the user can measure tongue bioimpedance (C). After the measurement is completed (D), tongue bioimpedance data is automatically processed by the UTES. Impedance data is sent from the UTES to the UTAP and data is automatically saved in the Android file system. Finally, the user can send all the data via email along with the user identifier, time-stamp, UTES firmware and UTAP software version (E, F). The operator can review the reconstructed longitudinal (in red) and transverse (in blue) conductivity and relative permittivity tongue data directly (G).

**FIGURE 5.**
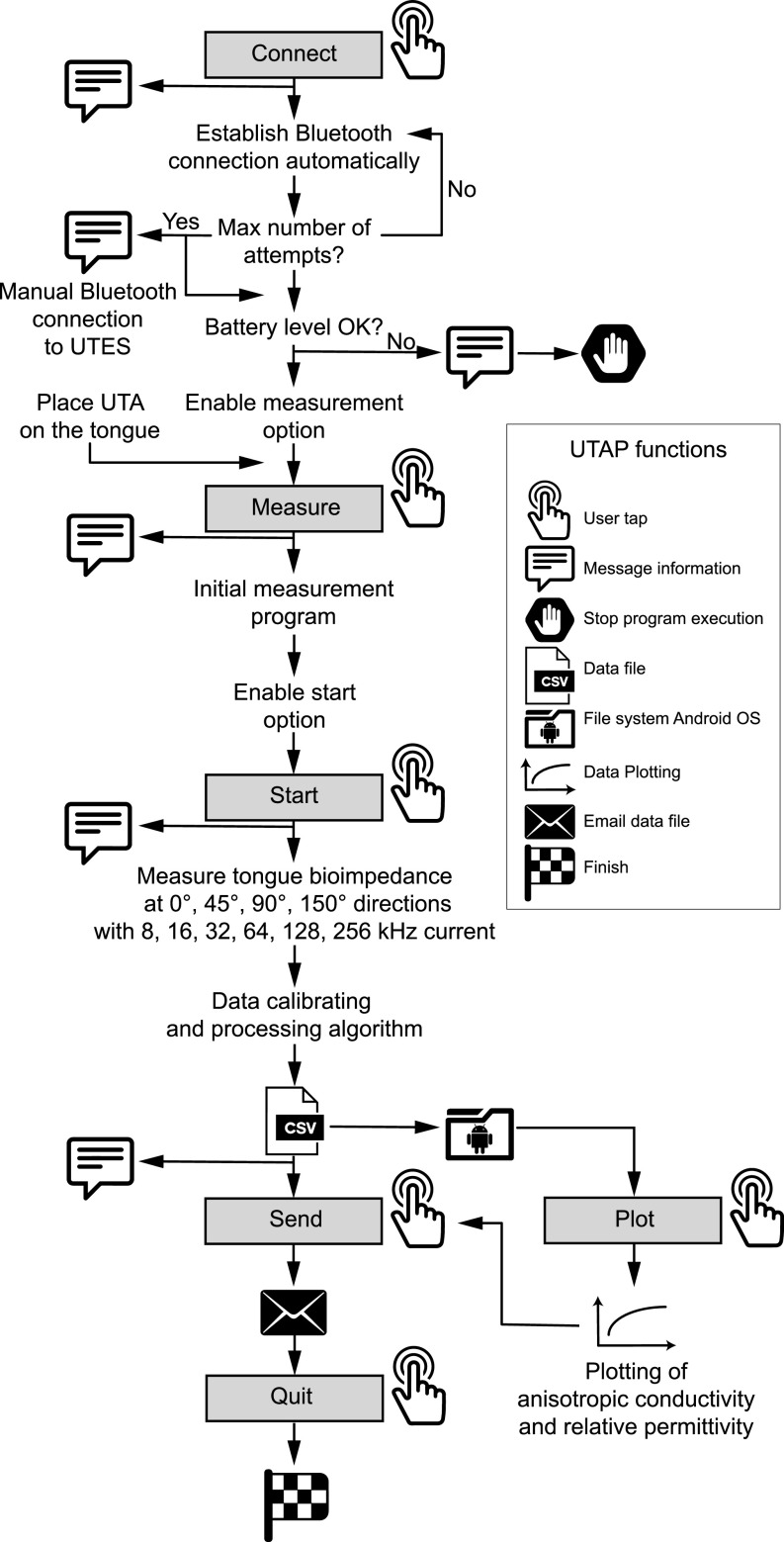
Flow for measuring tongue volume conduction properties (VCP) values. The operator can use the user tongue application (UTAP) to control the user tongue electronic system (UTES) and measure tongue VCP.

Figs. 5 and 4 show the flow diagram and the user interface of the Android user tongue application (UTAP) developed with Android Studio development environment and Java programming language. The UTAP connects to the user tongue electronic system (UTES) via Bluetooth. Two working modes in the UTAP are supported with conditional code compilation: *single*-user mode and *multi*-user mode. In the single-user mode, the patient identifier is entered only the first time the application is initialized and this information is then stored in the smartphone; whereas in the multi-user mode, this auto-save feature is disabled and the patient identifier must be entered every time the application is initialized. These two modes allow us to easily customize the application for two different purposes. In single-user mode, we aim to deploy our technology for patient at-home monitoring. The multi-user mode is intended for use in the clinic, where a single device will be used to collect data on different patients. In both modes, the operator identifier must also be entered each time to have a record of who actually performed the measurement.

The functionality of the UTAP shown in Fig. 5 includes: (1) automatic pairing with the UTES, (2) perform a tongue VCP measurement, (3) transfer the data, and (4) plot the data. When the user starts a new session and taps Connect button in the UTAP interface, the application connects to the UTES via Bluetooth automatically and stores the corresponding universally unique identifier (UUID) of the UTES. This feature provides an automated connection between the UTAP and UTES with no user effort. In case of Bluetooth pairing failure (e.g., the UTES power is off or the device is out of range), the operator has to manually pair both devices through the Bluetooth connection scanner. If the connection is successfully established between the UTES and the UTAP, the user can place the UTA depressor on the tongue and then tap the Measurement button to measure tongue VCP values. Once the user taps Start button, the tongue impedance is measured at each frequency for all four directions sequentially. De-identified user tongue data including the subject identifier, UIUD, battery level, date, start time are saved automatically in the Android operating system file system for backup. Data file also can be sent via email.

### D. Tongue Electrical Properties Emulator (TEPE)

**FIGURE 6.**
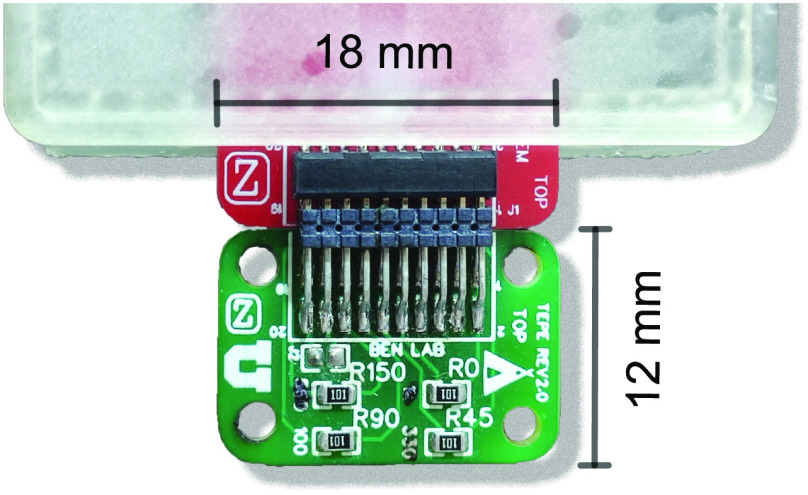
Tongue electrical properties emulator (TEPE) board for calibrating and testing the user tongue electronic system (UTES). The TEPE board has 4 pre-defined resistors for mimicking 4 directional tongue measurements and calibrate all the measurement channels in UTES.

The tongue electrical properties emulator (TEPE) is a printed circuit board (dimensions width 18 mm × length 12 mm inch) that connects to the UTES with surface mount soldered resistors (range 0.1 to 1.5 k$\Omega$) mirroring a wide range of healthy and diseased tongue VCP values (Fig. 6). UTES calibration is performed using a state-of-the-art calibration algorithm described elsewhere and takes only few seconds to complete [18]. The TEPE dummy circuit can also be used to test the correct functioning of the device, for example, prior to conducting a measurement on a subject.

### E. Experimental Protocol

This study was performed under Institutional Review Board approval at Beth Israel Deaconess Medical Center. Tongue UTA measurements were performed subjects participating in the clinical study “Innovative Measures of Speech and Swallowing Dysfunction in Neurological Disorders” (see clinicaltrials.gov NCT02118805 for further information). For all subjects recruited, measurements were performed after obtaining written informed consent. Subjects were asked to rinse their mouth with a small amount of water to ensure good electrical contact between the UTA and the tongue (see an example in Fig. 8). The UTA was centered on the tongue and data collected. For each healthy participant, the test took approximately 30 seconds to complete (see Supplementary Materials).

**FIGURE 7.**
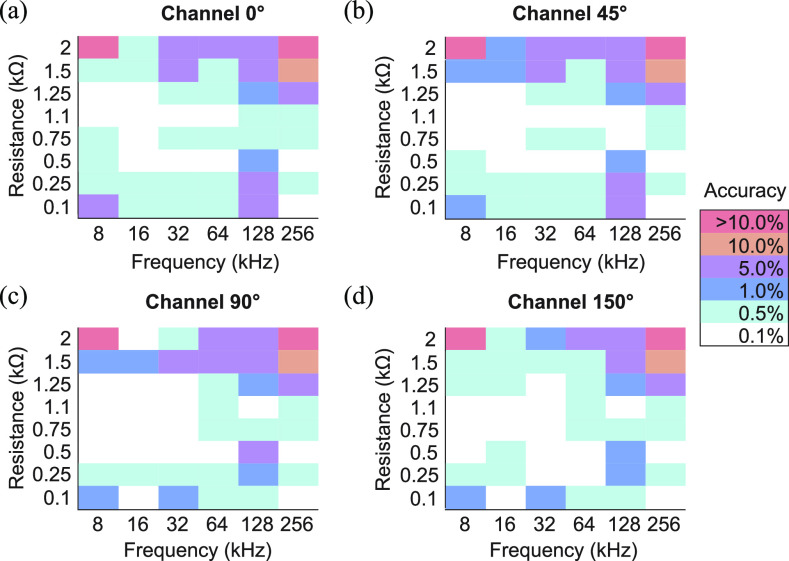
The chart presented indicates the user tongue electronic system (UTES) accuracy for given frequency and resistance values populated in tongue electrical properties emulator (TEPE) boards. In the area indicated in white color, a 0.1% accuracy is specified between 8 and 256 kHz with limitations towards larger resistance values and higher frequencies.

**FIGURE 8.**
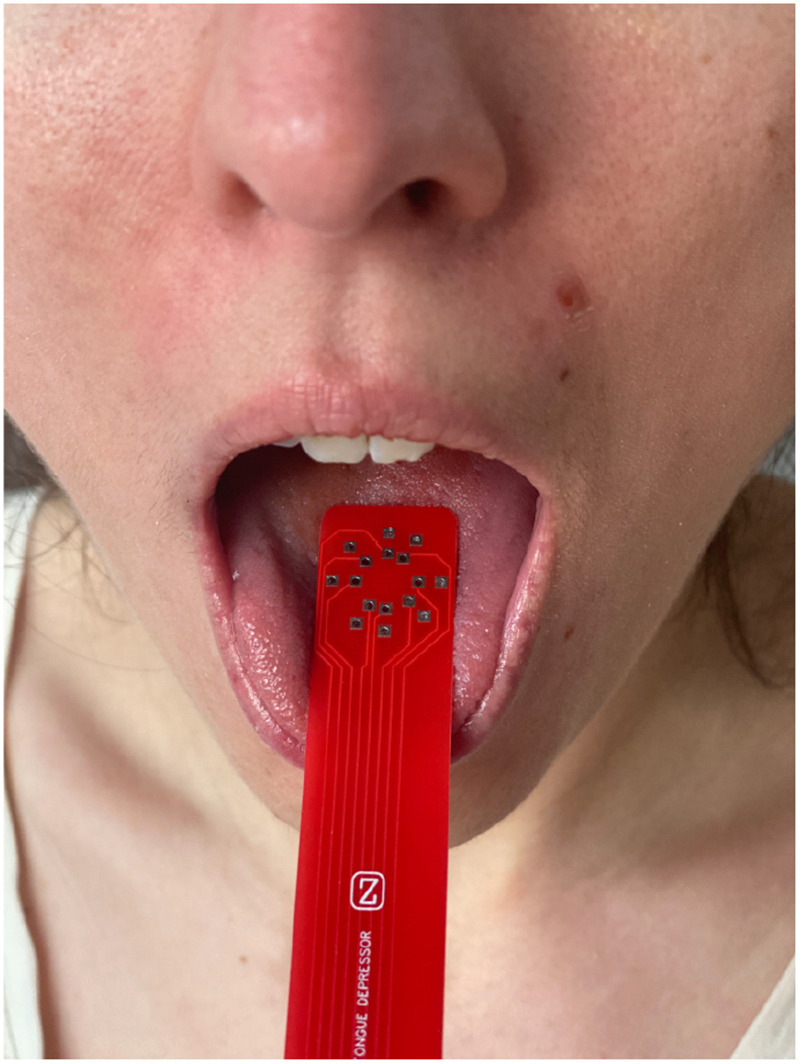
Use example of the UTES and UTA for measuring tongue electrical properties (TEP) in a healthy subject. The UTA depressor was sterilized by autoclave.

All measurements were made by the patients themselves while comfortably seated after being taught how to do so by the senior author (BS). The entire procedure took approximately 5 minutes per subject to complete.

### F. subject Information

We recruited 7 healthy subjects between ages 26 and 71 years. Exclusion criteria included history or family history of a generalized neuromuscular disease; history of recent trauma or other muscle injury; history of recent prolonged activity; generalized weakness or atrophy identified on examination; pregnant women; presence of a pacemaker or implanted cardiac defibrillator; presence of multiple simultaneous neuromuscular disorders; unstable psychiatric disease, cognitive impairment, or substance abuse; structural tongue abnormality; and tongue jewelry.

## III. Characterization Results

### A. Accuracy

Fig. 7 shows the accuracy of the UTES performing multifrequency measurements connected to TEPE boards populated with reference resistors. All four channels had resistors values ranging from 0.1 to 2 k$\Omega$ covering a broad range of tongue resistivity (i.e., inverse of conductivity) values. We refer the reader to Figure S1 in Supplementary Material for more details of UTES accuracy and signal to noise ratio. In vivo tongue impedance values measured with the UTA depressor range from 100 to 300 $\Omega$, where the precision of UTES is $< $1%.

## IV. Experimental Results

### A. Tongue Apparent Conductivity

**FIGURE 9.**
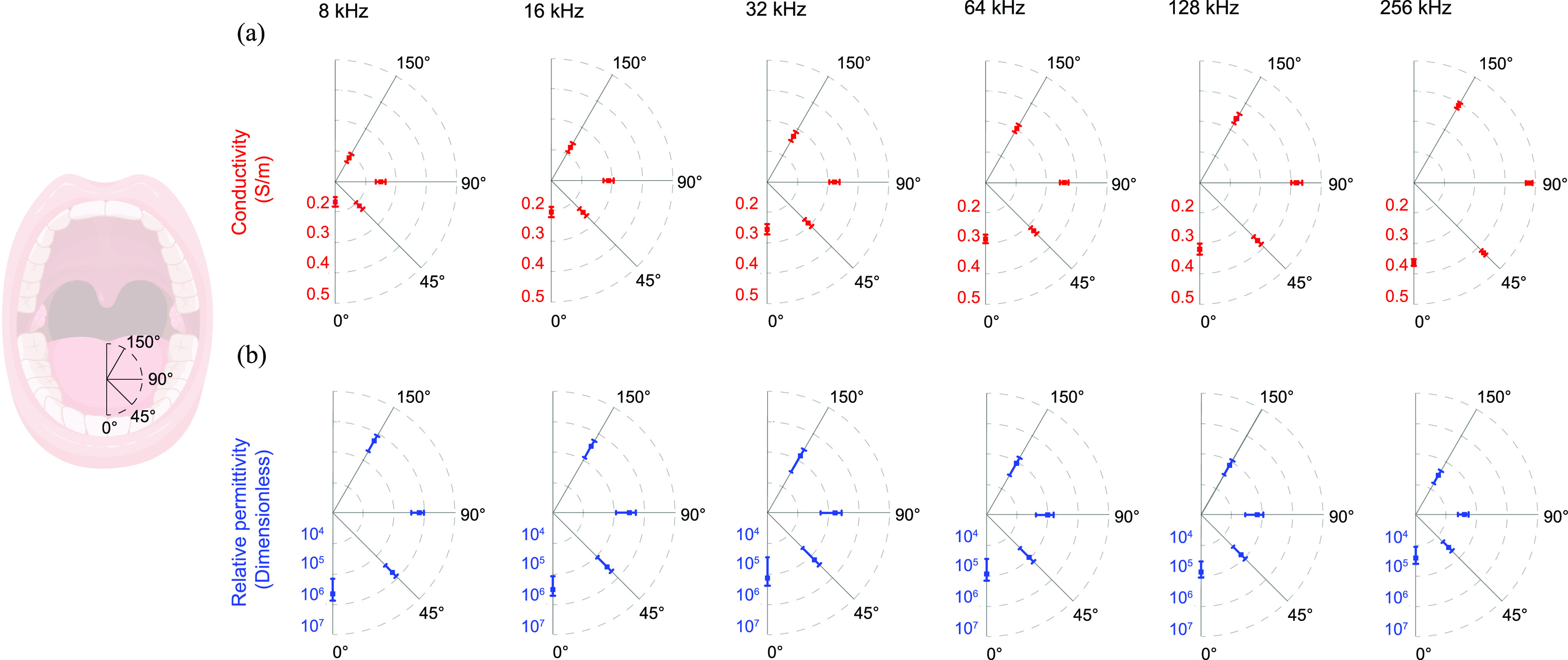
Anisotropy of tongue apparent conductivity (a) and relative permittivity (b) at (8, 16, 32, 64, 128 and 256) kHz. The direction determined by the user tongue array (UTA) angles 0$^{\circ }$ and 90$^{\circ }$ correspond to the sagittal and coronal tongue's planes. Mean $\pm$ standard error of the mean shown.

Fig. 9 (a) shows the spatial dependence of the tongue conductivity, a concept also known as anisotropy [21], [22]. In the sagittal plane defined by the 0$^{\circ }$ measuring direction, the conductivity is minimal at all frequencies, while in the transverse plane defined by the 90$^{\circ }$ measuring direction reaches the maximum value, thus supporting the observation that tongue is a highly anisotropic electrical tissue. As expected, the apparent conductivity increases with the frequency of electrical current. Apparent conductivity values shown in Fig. 9 are reported in Table I in Supplementary Information.

### B. Tongue Apparent Relative Permittivity

Fig. 9 (b) shows relative permittivity measured at direction 0$^{\circ }$, 45$^{\circ }$, 90$^{\circ }$, 150$^{\circ }$ at (8, 16, 32, 64, 128 and 256) kHz. Overall, the permittivity decreases with the frequency. In contrast to the conductivity, permittivity exhibits angular symmetry indicating no preferred directional dependence, probably due to the uniformity in the disposition of myofiber membranes in the measurement region. Apparent permittivity values shown in Fig. 9 are reported in Table II in Supplementary Information.

## V. Discussion

Here, we created a novel IoT-based device for future tongue VCP evaluation in patients with bulbar impairment. The test has the potential to be performed in the clinic or at home with minimal training, it is suitable for (young) children and adults, it requires minimal subject collaboration, and provides standard, quantitative and objective data reflecting tongue ionic composition and membrane integrity. This new IoT-device aims to be a reduced size, performance and cost version of our recent IoT high-performance platform [18], the former being able to be easily duplicated to allow the deployment for at-home studies. This last feature is especially important to address the challenge of FSHD patients visiting a study center on a regular basis to be part of a clinical study or treatment trial. Enabling at-home measurements will allow the patients to perform daily tongue measurements, today not currently possible with existing modalities. Finally, the intuitive smartphone software allows the study center to receive data through Internet for further medical analysis and interpretation.

Previous clinical studies have shown the potential value of electrical impedance myography (EIM) for following ALS progression with limb muscle measurements [23]. Basic measurements with surface electrodes in contact with the tongue have shown to correlate with standard measures thus suggesting that the examination of the tongue EIM could add valuable clinical information [24]–[26]. More recently, researchers from the University of Sheffield have shown the value of using more advanced EIM measuring approaches to enable ALS disease classification and correlation with disease severity [27]. However, these studies have in common that tongue EIM resistance and reactance values are not standardized outcomes and therefore the clinical adoption of these approaches as a standardized diagnostic tool or normative data is questionable [12], [28].

The electrical conduction properties of biological tissues and fluids have been studied almost continuously ever since suitable theory and adequate electrical techniques became available for this purpose [29]–[36]. A previous study revealed that these conduction properties provide significant contrast between healthy and diseased tissue types and strongly suggested that measuring tongue VCP in a clinical setting could be useful for tongue health assessment [37]. However, *in vivo* assessment of human tongue VCP has been only pursued in one study before [38]. In that work, the authors omitted the tongue's strong directional dependence which results in different conductivity values along and perpendicular to the tongue's main axis in the sagittal plane (see Fig. 9(a)). While valuable as a first effort, this rough approximation of tongue myoarchitecture questions the accuracy of the data reported [39]. To the best of the authors’ knowledge, this is the first time the anisotropy of tongue VCP has been studied including its dependence with the electromagnetic wave (i.e., frequency).

The apparent conductivity and the apparent relative permittivity are increasing and decreasing with the frequency, respectively, a result consistent with the Maxwell-Wagner interfacial polarization effect of the myofiber cellular membrane in the kHz frequency range [19], [20]. The dependence of the conductivity with the direction is determined by the complexity in the myoarchitecture of the tongue blade along the sagittal plane (i.e., longitudinal direction) and axial and coronal planes (i.e., transverse directions). The lack of anisotropy in the apparent relative permittivity values suggests no preferred direction to store electrical charges within the tongue. In patient with lingual impairment, tongue VCP are expected to reflect anomalous electrical and physical behavior of molecules including the myofiber membrane instability, changes in passive cell membrane capacitance, intracellular organelle membranes and protein molecule response.

This study has limitations. First, data are sent by the UTAP as a mail attachment, and thus the maximum number of measurements in one session is limited by the Gmail email server account to 20. Although we do not foresee this to be a problem, it is still possible to collect and send more data simply by starting a new session in the UTAP. Still, Gmail email server send limit is approximately 100-150 emails per day. While this has not been a limitation in this first study, in the future, we will consider other email platforms with larger email server send limit so as to evaluate our technology in larger study across the country measuring patients in multiple study centers. Another limitation are the electrodes created from Pb-free solder paste. Further research on new materials and coatings is warranted to lower the tongue-electrode polarization impedance affecting tongue measurements. This work represents our first effort in measuring tongue VCP in healthy subjects and, therefore, the number of subjects measured is limited. To evaluate clinical validity as a diagnostic biomarker, we need to include diseased patients and further increase the number of subjects measured to differentiate healthy from diseased subjects. We are currently working to evaluate the clinical utility of our technology by measuring patients with FSHD and patients with tongue cancer with studies being conducted at the University of Utah and at the MD Anderson Cancer Center in Houston, TX. As part of these research efforts, we will characterize the technical performance of tongue VCP data as a biomarker including its sensitivity and specificity in these conditions. Further work we plan to pursue includes to determine tongue VCP reproducibility with measurements made by the patients themselves at-home.

Despite the limitations, IoT UTA device can help overcome challenges to recruitment and retention of patients with bulbar impairment in clinical studies and help reduce costs and accelerate research outcomes in devastating disease such ALS and FSHD. In ALS specifically, as the disease progresses, most voluntary muscles experience continuous fatigue ultimately leading to paralysis at a late stage of the disease. IoT-powered at-home evaluation can help overcome these important practical challenges in future clinical studies having a direct impact on patient care.

## VI. Conclusions

The UTA based on IoT technology can improve tongue evaluation in clinical and at-home trials. With minimal training, patients will be able to perform frequent measurements on themselves more frequently thus reducing the necessary sample size for a longitudinal study in order to achieve statistical power to detect disease progression and therapeutic effect [40]. Bringing the UTA technology in drug trials has the potential to lower the costs of clinical research and, ultimately, speed up the invention and validation of new therapies to a broad range of neurological disorders affecting speech and swallowing.

## VII. Supplementary Materials

The supplementary materials include the algorithm to compute tongue VCP values (Part A), UTES characterization results measuring phantom circuits (Part B: Figure S1), and the apparent tongue VCP data (Part C: Tables I and II).

Supplementary materials

## Conflict of interest

Dr. Sanchez holds equity in Haystack$^{\rm Dx}$, a company that develops clinical needle impedance technology for neuromuscular evaluation. The company has an option to license patented needle impedance technology where the author is named an inventor. He also holds equity and serves as Scientific Advisory Board Member of Ioniq Sciencies, a company that develops clinical impedance technology for early cancer detection. Dr. Sanchez serves as Scientific Advisory Board Member of B-Secur, a company that develops wearable ECG and impedance technology. He consults for Myolex, Inc., a company that develops surface impedance technology. The company has an option to license patented surface EIM technology where the author is named an inventor. Dr. Sanchez also serves as a consultant to Impedimed, a company that develops clinical impedance technology for early detection of secondary lymphedema, and Texas Instruments, Happy Health, and Maxim Integrated, companies that develop impedance related technology for consumer use. This study did not employ any relevant company technology.
