# Supplementary material for: A Bioimpedance-Based Device to Assess the Volume Conduction Properties of the Tongue in Neurological Disorders Affecting Bulbar function
Source: IEEE Open J Eng Med Biol. 2021 Oct 6;2:278–85. doi: 10.1109/OJEMB.2021.3117871 (PMC8940204; doi:10.1109/OJEMB.2021.3117871)
Supplement: Supplementary materials [file supp1-3117871.pdf]

Supplementary Materials

A bioimpedance-based device to assess the volume conduction properties of the tongue in neurological disorders affecting bulbar function

Xuesong Luo, Hilda V. Gutierrez Pulido, Seward B. Rutkove, *IEEE Member*, Benjamin Sanchez, *IEEE Senior Member*

I. Description

THE supplementary materials provide additional information of our work. Part A provides additional algorithm details implemented in the device presented to compute tongue volume conduction properties (VCPs). Part B consists of Figure S1, that shows the device's accuracy results measuring the resistors in tongue electrical properties emulator (TEPE) at 128 kHz. Part C contains the tongue VCP data reported in the text.

II. Part A: Data processing algorithm

To calculate apparent tongue electrical property values, we first build matrices $\mathbf{R}$ and $\mathbf{X}$ with size 4×4 and $\mathbf{I}$ a column-wise vector with size 4×1, defined as

$$\mathbf{R} := \begin{bmatrix} R^2(0^\circ) & 0 & 0 \\ R^2(45^\circ)/2 & R^2(45^\circ)/2 & R^2(45^\circ)/4 \\ 0 & R^2(90^\circ) & 0 \\ 3R^2(150^\circ)/4 & R^2(150^\circ)/4 & -\sqrt{3}R^2(150^\circ)/4 \end{bmatrix},$$

$$\mathbf{X} := \begin{bmatrix} X^2(0^\circ) & 0 & 0 \\ X^2(45^\circ)/2 & X^2(45^\circ)/2 & X^2(45^\circ)/4 \\ 0 & X^2(90^\circ) & 0 \\ 3X^2(150^\circ)/4 & X^2(150^\circ)/4 & -\sqrt{3}X^2(150^\circ)/4 \end{bmatrix},$$

and $\mathbf{I} := [1\ 1\ 1\ 1]^\top$, where $\top$ is the transpose operator. The next step is to compute the least square solution $\mathbf{G} = (\mathbf{R}^\top\mathbf{R})^{-1}\mathbf{R}^\top\mathbf{I}$ and $\mathbf{B} = (\mathbf{X}^\top\mathbf{X})^{-1}\mathbf{X}^\top\mathbf{I}$ where $\mathbf{G} := [G_1\ G_2\ G_3]^\top$ and $\mathbf{B} := [B_1\ B_2\ B_3]^\top$. Next, we can define the following quantities

$$g_1 := \frac{(G_1+G_2)+\sqrt{(G_1-G_2)^2+G_3^2}}{2}, b_1 := \frac{(B_1+B_2)+\sqrt{(B_1-B_2)^2+B_3^2}}{2}$$

and

$$g_2 := \frac{(G_1+G_2)-\sqrt{(G_1-G_2)^2+G_3^2}}{2}, b_2 := \frac{(B_1+B_2)-\sqrt{(B_1-B_2)^2+B_3^2}}{2}$$

Then the longitudinal (L) and transverse (T) tongue conductivity σ (S m^{-1}) and relative permittivity ε (dimensionless) can be calculated as follows

$$\begin{cases} \sigma_L := \frac{\rho_L}{\rho_L^2+\tau_L^2} & \text{and} \quad \sigma_T := \frac{\rho_T}{\rho_T^2+\tau_T^2} \\ \varepsilon_L := \frac{1}{2\pi f\varepsilon_0}\frac{-\tau_L}{\rho_L^2+\tau_L^2} & \text{and} \quad \varepsilon_T := \frac{1}{2\pi f\varepsilon_0}\frac{-\tau_T}{\rho_T^2+\tau_T^2} \end{cases}$$

with $\rho_L = \sqrt{g_2}/g_1$, $\rho_T = 1/\sqrt{g_2}$, $\tau_L = -\sqrt{b_2}/b_1$, $\tau_T = -1/\sqrt{b_2}$ and $\varepsilon_0 = 8.85\cdot 10^{-12}$ (F m^{-1}). Finally, we can obtain the apparent conductivity $\sigma_a(\theta)$ (S m^{-1}) and relative permittivity $\varepsilon_a(\theta)$ (dimensionless)

$$\sigma_a(\theta) = \sqrt{\sigma_L\sigma_T}\sqrt{\cos^2\theta + \frac{\sigma_L}{\sigma_T}\sin^2\theta}$$
$$\varepsilon_a(\theta) = \sqrt{\varepsilon_L\varepsilon_T}\sqrt{\cos^2\theta + \frac{\varepsilon_L}{\varepsilon_T}\sin^2\theta} \qquad \text{(S1)}$$

where $\theta \in \{0^\circ, 45^\circ, 90^\circ, 150^\circ\}$ is the angle of measuring direction. We refer the reader to [1], [2] for further details.

III. Part B: Characterization results

Figure S1 A shows an example of continuous measurements in all four channels at 128 kHz. The non-steady impedance response at the beginning of each measurement is caused by the transient response of the internal low pass filter built-in the AFE4300 circuit. To avoid transient errors affecting the accuracy of our measurement, the UTES runs a real-time algorithm that allows automated steady-state detection in all channels. The signal-to-noise ratio (SNR) is then calculated from the estimated values of mean and standard deviation (Figure S1 B).

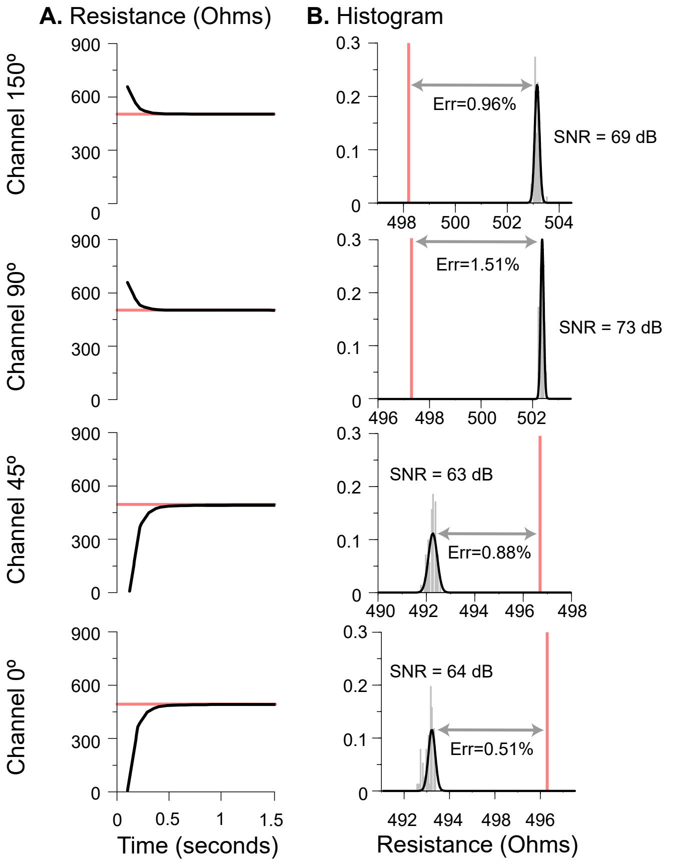

Fig. S1. Continuous (A) and histogram (B) resistance measured at 128 kHz using the tongue electrical properties emulator (TEPE). Channel 0°, 45°, 90°, 150° of user tongue electronic system (UTES) are configured to measure the TEPE board populated with reference resistances (in red). The amplitude distribution is plotted for each channel to determine the error and signal to noise ratio (SNR).

IV. Part C: Experimental results

Tables I and II provide tongue VCP values shown in Figure 9 measuring N=7 healthy subjects.

Tongue apparent conductivity (S/m)

Freq (kHz)	Anisotropic conductivity (S/m) 0°	45°	90°	150°	Avg SD
8	0.166	0.212	0.249	0.190	0.016
16	0.203	0.247	0.288	0.225	0.017
32	0.255	0.290	0.321	0.273	0.017
64	0.285	0.324	0.358	0.305	0.014
128	0.318	0.368	0.419	0.343	0.018
256	0.361	0.424	0.479	0.393	0.011

TABLE I. Apparent tongue conductivity values at (8, 16, 32, 64, 128 and 256) kHz measured at 0°, 45°, 90°, 150°. Mean±averaged standard deviation (Avg SD) are reported.

Tongue apparent relative permittivity

Freq (kHz)	Anisotropic relative permittivity (dimensionless) 0°	45°	90°	150°	Avg SD
8	455538	584728	690148	524128	309171
16	328748	324588	324769	326015	206316
32	137498	151343	164074	144577	108644
64	86227	93113	99597	89723	58261
128	75421	71972	69182	73614	41085
256	26652	33536	39261	30283	15120

TABLE II. Apparent tongue relative permittivity values at (8, 16, 32, 64, 128 and 256) kHz measured at 0°, 45°, 90°, 150°. Mean±averaged standard deviation (Avg SD) are reported.

References

[1] H. Kwon, J. Nagy, R. Taylor, S. Rutkove, and B. Sanchez, "New electrical impedance methods for the in situ measurement of the complex permittivity of anisotropic biological tissues," *Physics in Medicine & Biology*, vol. 62, no. 22, p. 8616, 2017.

[2] H. Kwon, M. Guasch, J. Nagy, S. Rutkove, and B. Sanchez, "New electrical impedance methods for the in situ measurement of the complex permittivity of anisotropic skeletal muscle using multipolar needles," *Scientific reports*, vol. 9, no. 1, pp. 1–16, 2019.
